# A eukaryotic initiation factor 5C is upregulated during metamorphosis in the cotton bollworm, *Helicoverpa armigera*

**DOI:** 10.1186/1471-213X-9-19

**Published:** 2009-03-08

**Authors:** Du-Juan Dong, Jin-Xing Wang, Xiao-Fan Zhao

**Affiliations:** 1 The Key Laboratory of Plant Cell Engineering and Germplasm Innovation of Ministry of Education, School of Life Sciences, Shandong University, Jinan 250100, Shandong, PR China

## Abstract

**Background:**

The orthologs of eukaryotic initiation factor 5C (eIF5C) are essential to the initiation of protein translation, and their regulation during development is not well known.

**Results:**

A cDNA encoding a polypeptide of 419 amino acids containing an N-terminal leucine zipper motif and a C-terminal eIF5C domain was cloned from metamorphic larvae of *Helicoverpa armigera*. It was subsequently named *Ha-eIF5C*. Quantitative real-time PCR (QRT-PCR) revealed a high expression of the mRNA of *Ha-eIF5C *in the head-thorax, integument, midgut, and fat body during metamorphosis. Immunohistochemistry suggested that Ha-eIF5C was distributed into both the cytoplasm and the nucleus in the midgut, fat body and integument. *Ha-eIF5C *expression was upregulated by 20-hydroxyecdysone (20E). Furthermore, the transcription of *Ha-eIF5C *was down regulated after silencing of *ecdysteroid receptor (EcR) *or *Ultraspiracle protein (USP) *by RNAi.

**Conclusion:**

These results suggested that during metamorphosis of the cotton bollworm, *Ha-eIF5C *was upregulated by 20E through the EcR and USP transcription factors.

## Background

To holometabolous insects, molting is a common physiological process, whose life cycles are characterized by a series of molts. During their larval molts, the larvae progress from one instar to the next. Thereafter, pupation and eclosion ensue during their metamorphic molts. Increasing evidence indicates that some hormones and receptors may contribute to the complex developmental pathways associated with molting and metamorphosis. Many genes have been shown to be involved in molting or metamorphosis, such as the transcription factors *ecdysteroid receptor (EcR)*, *Ultraspiracle protein (USP)*, *Hormone receptor 3 (HR3) *and *Broad complex *[1], and the programmed cell death pathway genes [2]. Some key regulatory genes have also been identified, such as *E74B *and *E93 *[3]. However, very few genes downstream of *Broad complex*, *E74B *and *E93 *have been identified. Consequently, there is a dearth of available knowledge on the molecular mechanisms that lead to larval molt and metamorphosis. By conducting a research of the molting related genes, we may further understand the molecular mechanism of development and ecdysone regulation, and find the novel molecular targets to effectively control the pest.

Suppression subtractive hybridization (SSH) is a useful method for identifying differentially expressed genes during larval molting. Using the metamorphically committed larvae (6th-72, 96 and 120 h) as the tester and the feeding 5th instar larvae (5th-24 h) as the driver, we obtained an EST, which was similar to *basic leucine zipper *by BLASTX analysis [4]. We designed primers based on this fragment to obtain the full-length cDNA and identified it as translation initiation factor 5C (*eIF5C*).

The regulation of translation plays an important role in the control of gene expression. In eukaryotes, translation regulation occurs primarily during the initial step, which is rate limiting under most circumstances [5]. More and more evidence suggests that translation initiation factors (eIFs) are not only essential in the initiation of protein translation but also important in other life processes. Some eIFs are regulators of signaling pathways, such as eIF4A of *Drosophila melanogaster*, which functions as a negative regulator of Dpp/BMP (decapentaplagic/bone morphogenetic protein) signaling that mediates SMAD (mother against dpp) degradation [6]. Eukaryotic initiation factor 6 selectively regulates Wnt signaling and β-catenin protein synthesis [7].

eIF5C is a phylogenetically conserved protein, which is said to contain an N-terminal leucine zipper motif and a C-terminal eIF5C domain. Our BLASTX results showed that homologs of *eIF5C *exist in various organisms, from *Cryptococcus neoformans *to *Homo sapiens*. BZAP45, the ortholog of eIF5C in humans, contributes to transcriptional control at the G1/S phase transition [8]. In *Rattus norvegicus*, brain development-related molecule 2 (*Bdm2*) is a developmentally regulated gene, which is highly expressed in fetal rat brain [9]. Wang *et al*. [10] showed that eIF5C was associated with the ribosome through an interaction with *D. melanogaster *ribosomal protein L5 (dRPL5), suggesting its possible role during protein synthesis in fruit flies. Given that there are no related functional reports to date, the information of eIF5C from other insects have been obtained from gene sequencing.

In this study, we cloned and characterized the eIF5C from the metamorphic larvae of *H. armigera *and designated it as *Ha-eIF5C*, which contains an N-terminal leucine zipper motif and a C-terminal eIF5C domain. The expression, distribution and characterization of Ha-eIF5C were studied by employing Quantitative real-time PCR (QRT-PCR), recombinant expression and immunoblotting analysis. Likewise, we also investigated the gene's hormonal regulation and its position in the 20E signal transduction pathway.

## Results

### Gene cloning and sequence analysis of Ha-eIF5C

**Figure 1:**
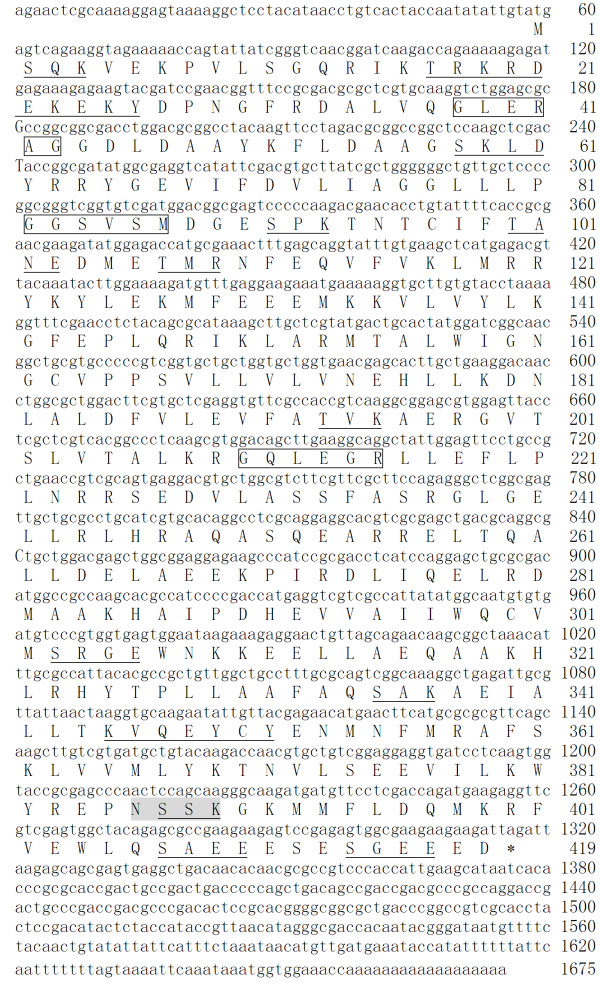
**Complete cDNA sequence and deduced amino acid sequence of *Ha-eIF5C***. The underlined amino acid sequences indicate predicted phosphorylation sites. Protein kinase C phosphorylation sites (2–4; 17–19; 91–93; 107–109; 193–195; 335–337; 387–389); tyrosine kinase phosphorylation sites (18–26; 345–351); casein kinase II phosphorylation sites (58–61; 100–103; 303–306; 407–410; 414–417). The putative N-glycosylation site is shaded. Predicted N-myristoylation sites are in block.

Based on the fragment of *Ha-eIF5C *obtained from suppression subtractive hybridization (SSH), the 5' end fragment was obtained using specific reverse primer eIF5CR and the T3 primer. The 3' end fragment was amplified with the specific primer eIF5CF and the T7 primer. The full-length *eIF5C *of *H. armegera *(1675 bp) was obtained through an assemblage of overlapping nucleic acids. This included a 57 bp 5' untranslated region (UTR), a 1260 bp open reading frame and a 340 bp untranslated region in the 3' UTR, with a 18 bp poly A tail. The ORF encoded a 419 amino acid protein with a calculated molecular mass of 48 kDa and a predicated isoelectric point of 6.05. Moreover, there were some putative post-translational modification sites including seven protein kinase c phosphorylation sites, two tyrosine kinase phosphorylation sites, three N-myristoylation sites, five casein kinase II phosphorylation sites and one N-glycosylation site (Fig. 1).

### Identification of *Ha-eIF5C*

**Figure 2:**
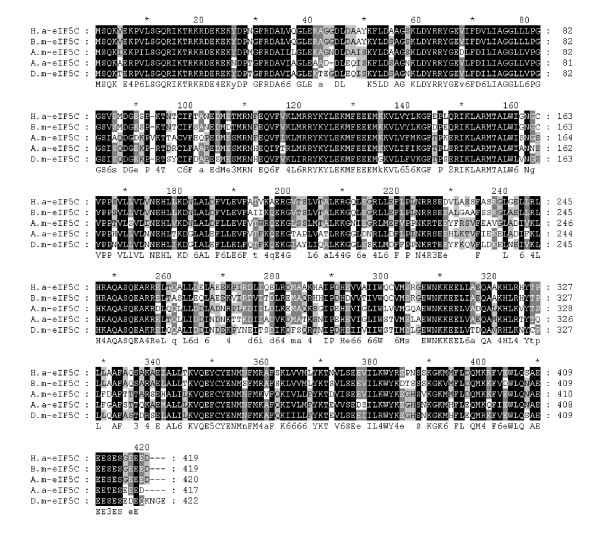
**Multiple alignments of Ha-eIF5C with other insect eIF5C**. eIF5C from *A. aegypti *XP_001655819, *A. mellifera *XP_395256, *B. mori *NP_001091797 and *D. melanogaster *Q9VNE2. The numbers on the right indicate the amino acid position of different sequences. Identical amino acids are shaded in black. Other conserved, but not consensus amino acids, are shaded in grey.

The result of the BLASTX analysis suggests that Ha-eIF5C has certain similarities to various genes, including *eIF5C *from *Bombyx mori *(88%), *eIF5C *from *Apis mellifera *(68%), *eIF5C *from *Aedes aegypti *(66%) and *eIF5C *from *D. melanogaster *(63%) (Fig. 2). SMART predicted that Ha-eIF5C protein contains a C-terminal eIF5C domain (326–411 aa) and an N-terminal leucine zipper motif (39–60 aa).

### Recombinant expression and purification of Ha-eIF5C

**Figure 3:**
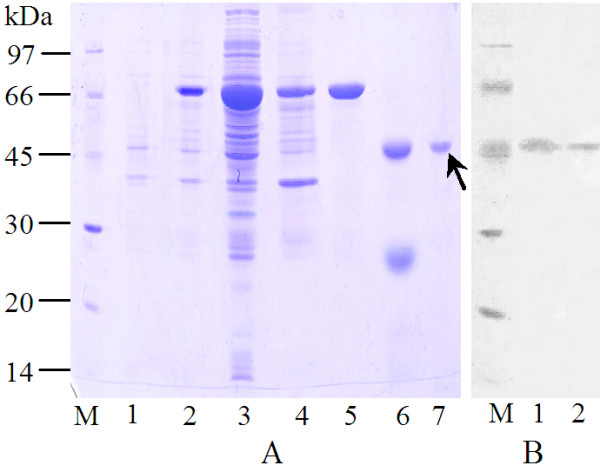
**Recombinant expression of Ha-eIF5C in *E. coli *and the preparation of anti-Ha-eIF5C antiserum**. A: SDS-PAGE analysis of recombinantly expressed Ha-eIF5C in *E. coli*. Lane 1, non-induction control; lane 2, induced expression of the fusion protein by IPTG; lane 3, supernatant after sonicating; lane 4, pellet after sonicating; lane 5, purified recombinant GST-eIF5C by Glutathione Sepharose 4B; lane 6, cleavage of GST-eIF5C with thrombin for 16hrs; lane 7, gel extraction of recombinant eIF5C. B: Immunoblotting showing the detection of purified recombinant eIF5C and the tissue extracts with the antibody. Lane 1, purified recombinant Ha-eIF5C without GST tag; lane 2, tissue homogenates from 6th instar 120 h larvae. M: standard protein; 12.5% SDS-PAGE.

After IPTG induction, the recombinant GST-eIF5C was expressed in supernatant and purified by Glutathione Sepharose 4B. The deduced molecular weight of the recombinant expressed protein was 48 kDa as shown in Fig. 3. To prepare the antiserum, a gel extraction of recombinant eIF5C after cleavage of GST-eIF5C with thrombin was used.

### Tissue distribution and expression patterns of Ha-eIF5C

**Figure 4:**
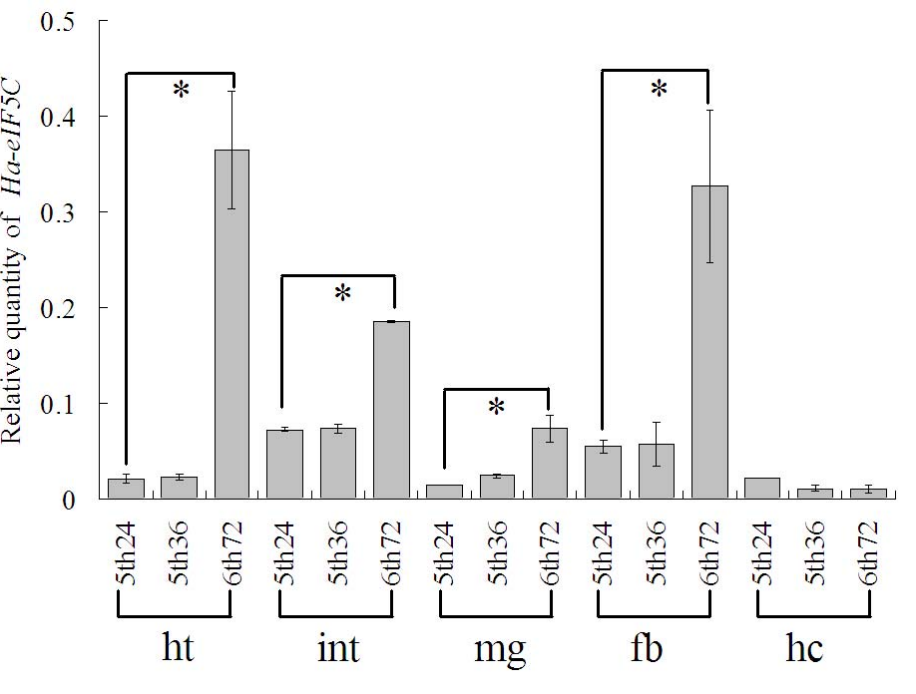
**QRT-PCR analysis of gene expression in five tissues during larval feeding, molting and metamorphosis**. ht: head-thorax, int: integument, mg: midgut, fb: fat body, hc: haemocytes. 5th24 = 5th instar larvae 24 h after ecdysis, 5th36 = 5th instar larvae 36 h after ecdysis (5th-HCS, with head capsule slippage), 6th72 = 72 h after ecdysis (wanderting 0 d, metamorphically committed larvae). Error bars represent the standard deviation in three replicates. An asterisk indicates significant differences (Student' s t-test, *: p < 0.05).

**Figure 5:**
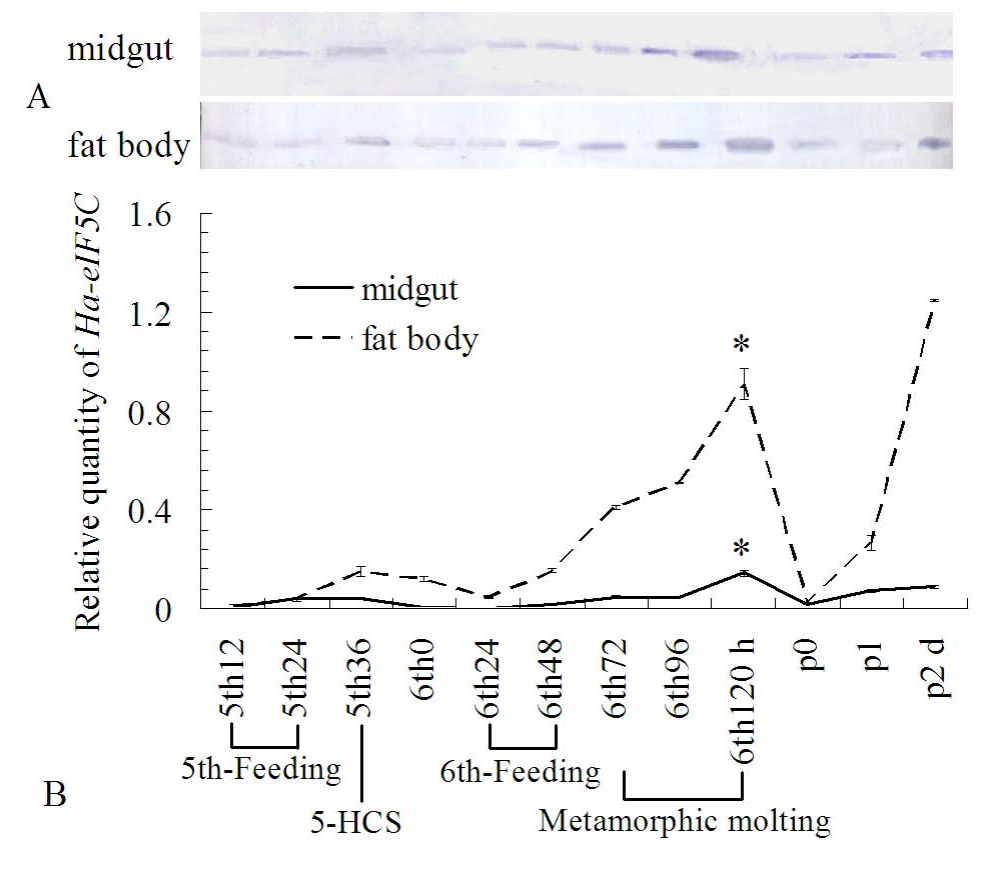
**Developmental expression of Ha-eIF5C in fat body and midgut showed by immunoblotting (A) and QRT-PCR (B)**. A: Immunoblotting showing the expression profiles of Ha-eIF5C protein in the midgut and fat body; B: QRT-PCR analysis of *Ha-eIF5C *mRNA transcription in the midgut and fat body from different developmental periods. 5th-Feeding: 5th instar larvae feeding stage; 5-HCS: 5th instar larvae with head capsule slippage; 6th-0: 6th instar 0 h larvae, white head (within 1 h after ecdysis); 6th-Feeding: 6th instar larvae feeding stage; Metamorphic molting: 6th instar metamotphic molting stage; p0: 0 h pupae; p1: first day pupae; p2: second day pupae. Error bars represent the standard deviation in three replicates. An asterisk indicates that the expression of *Ha-eIF5C *at 6th instar 120 h had statistically significant differences from those at 5th and 6th feeding stages (Student' s t-test, *: p < 0.05).

To study the tissue distribution of *Ha-eIF5C*, the total RNA of the head-thorax, integument, midgut, fatbody and haemocyte were extracted from 5th 24 h (5th instar larvae 24 h after ecdysis), 5th-HCS (5th instar larvae 36 h after ecdysis, with head capsule slippage, HCS) and 6th 72 h (72 h after ecdysis, wandering 0 d, metamorphically committed larva) stage. As shown in Fig. 4, *Ha-eIF5C *transcript was detected at a high level in the head-thorax, integument, midgut and fat body but not in haemocytes in metamorphosis stage. QRT-PCR was utilized to analyze the expression of *Ha-eIF5C *in developmental midgut and fat body. The results showed that there was an obvious increase in the level of *Ha-eIF5C *transcript during metamorphosis. The immunoblotting revealed that the expression of Ha-eIF5C protein agreed with the mRNA transcription (Fig. 5).

### Hormonal regulations on Ha-eIF5C

**Figure 6:**
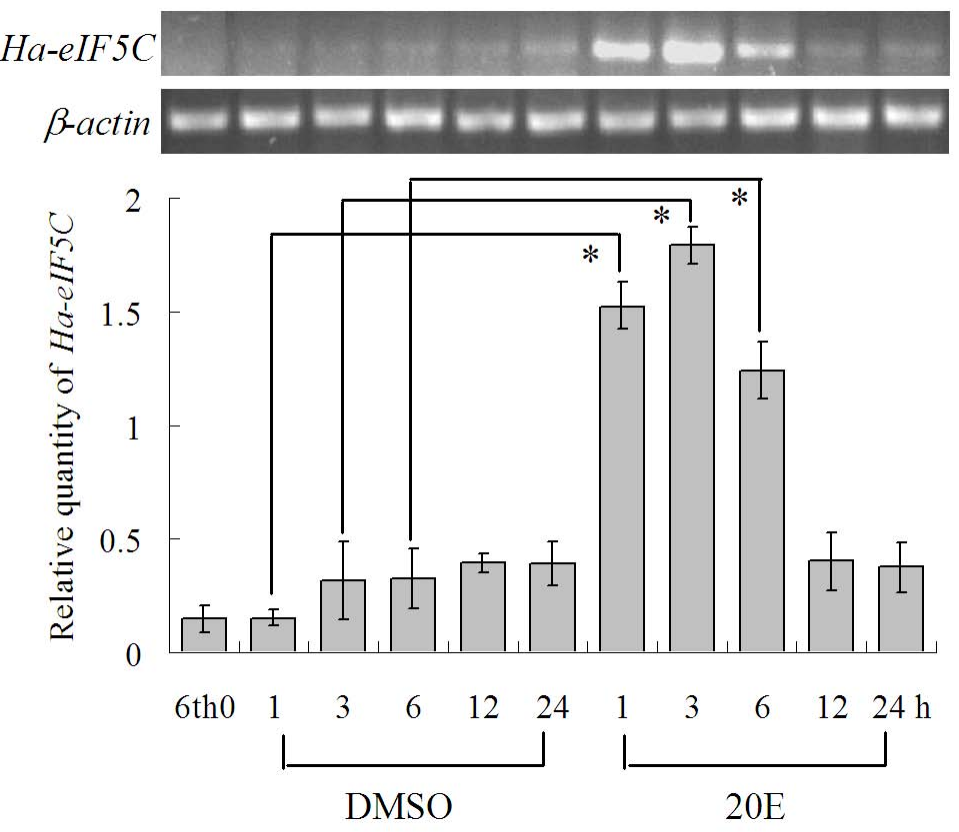
**20-hydroxyecdysone regulation of *Ha-eIF5C *expression in the midgut detected by semiquantitative RT-PCR analysis**. 6th instar 0 h larvae were injected with 20E (0.5 μg/larva), DMSO as control. 6th 0: normal 6th instar 0 h larvae; 1, 3, 6, 12 and 24 are durations (hour) after the injection of 20E. β-*actin *is used as a quantitative control. Error bars represent the standard deviation in three replicates. An asterisk indicates significant differences (Student' s t-test, *: p < 0.05).

To examine the effect of ecdysone on Ha-eIF5C expression, 6th instar 0 h larvae (6th-0 h, with white head capsule) were injected with 20-hydroxyecdysone (20E). Compared with the control, a 5–6-fold increase in *Ha-eIF5C *expression was observed at 1 h and 3 h after the challenge. However, the expression level of *Ha-eIF5C *started to decline at 6 h and returned to the basal level at 12 h (Fig. 6).

**Figure 7:**
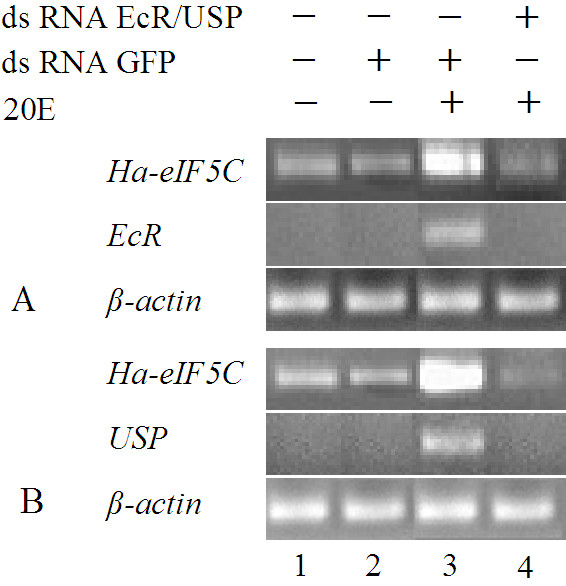
**Down-regulation of *Ha-eIF5C *expression upon RNAi knock down of EcR (A) or USP (B) in the HaEpi cell line, 12 h induction by 20E**. Lane1, normal cells without treatment; Lane2, cells treated with dsRNA of GFP; Lane3, cells treated with dsRNA of GFP and 20E; Lane4, cells treated with dsRNA of EcR/USP and 20E.

In order to study whether *Ha-eIF5C *was upregulated downstream of the 20E-induced transcription cascade, we knocked down EcR and USP in the HaEpi cell line by RNAi. After either EcR or USP was knocked down via RNAi, the transcription of *Ha-eIF5C *was down regulated compared with the control and it could not be upregulated anymore by treatment with 20E (Fig. 7).

### Immunohistochemistry

**Figure 8:**
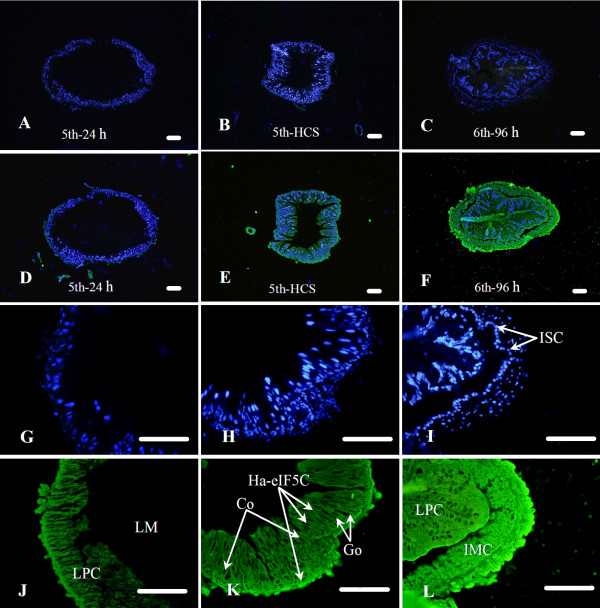
**Immunocytochemical localization of eIF5C in the midgut**. Panels A-C are negative controls with pre-immune rabbit serum; panels D-F are midgut from feeding 5th instar larva (5th-24 h), molting 5th instar larva (5th-HCS) and 6th-96 h (wandering) larva; panels G and J, H and K, I and L are the magnified D, E, F, respectively; nuclear staining was done by DAPI (G, H, I) and the positive signals were detected by ALEXA 488 assay (J, K, L), panels A-F are overlay. LM, lumen of midgut; LPC, larval polyploidy cells; ISC, intestinal stem cell; IMC, imaginal cells; Co, clumnar cells; Go, goblet cells. Scale bar = 100 μm.

**Figure 9:**
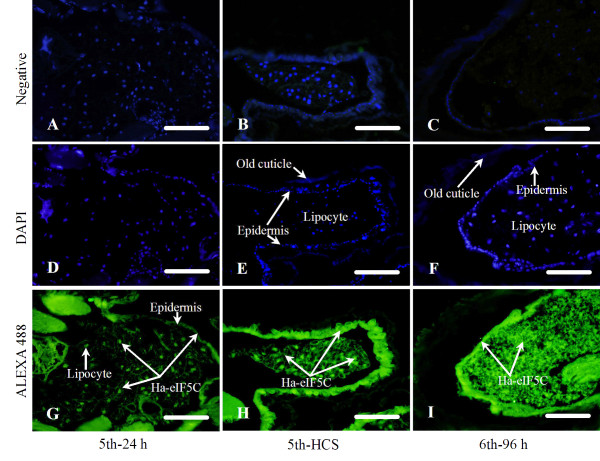
**Immunocytochemical localization of eIF5C in the integument and fat body**. Panels A-C are negative controls with pre-immune rabbit serum (merged into DAPI staining outcome); panels D-I are integuments and fat body from feeding 5th instar larvae (5th-24 h, D&G), molting 5th instar larvae (5th-HCS, E&H) and 6th-96 h larvae (wandering, F&I). The nuclei were stained with DAPI (D, E, F) and the positive signals were detected using ALEXA 488 as the secondary antibody (G, H, I). Scale bar = 100 μm.

To verify the expression and localization of Ha-eIF5C, we performed an immunohistochemical analysis of the midgut (Fig. 8), fat body and integument (Fig. 9) from feeding 5th larvae (5th-24 h), molting 5th larvae (5th-HCS) and wandering 6th-96 h larvae (6th-96 h). In the 5th-HCS stage, the midgut epithelium consisted of larval polyploid cells (LPC, including columnar and goblet cells) and intestinal stem cells (ISC) (Fig. 8-K). Larval ISCs are the progenitors of the adult midgut epithelium. The larval polyploid cells moving into the lumen from the basement membrane were replaced by proliferating and differentiating ISCs at 6th-96 h (Fig. 8-L). At this point, groups of imaginal cells began to form cell layers. Our immunohistochemical analysis shows that Ha-eIF5C was distributed into both the cytoplasm and nucleus in the midgut during the feeding 5th, molting 5th and wandering 6th-96 h stages. Relatively strong fluorescent signals were detected on the outer peripheries of the midgut epithelium from worms during 5th instar feeding and molting stage, as well as the larval polyploid cells and the imaginal cells from larvae at wandering stage. Likewise, ISCs, muscle cells and basement membrane were localized in this area.

At the same time, the localization of Ha-eIF5C in the integument and fat body was detected by immunohistochemistry. During the molting 5th and wandering 6th-96 h (Metamorphic molting), a cascade of physiological processes occurred. These included the separation of the old cuticle from the underlying epidermis, followed by the secretion of a new cuticle beneath the old. Finally, a shedding of the old exoskeleton occurred (Fig. 9-E, F). It was obvious that Ha-eIF5C appears localized in both cytoplasm and nucleus in the epidermis and lipocyte.

## Discussion

In this work, we identify a 1675 bp full-length elF5C from *H. armegera*. This includes a 1260 bp open reading frame encoding a 419 amino-acid protein with a predicted molecular mass of 48 kDa. Protein alignments showed that Ha-eIF5C and eIF5C from *A. aegypti*, *A. mellifera*, *D. melanogaster *and *B. mori *are very similar.

Ha-eIF5C is a phylogenetically conserved protein predicted to contain an N-terminal leucine zipper motif (39–60 aa) and a C-terminal eIF5C domain (326–411 aa). This eIF5C domain was first detected at the very C-termini of the yeast protein GCD6, eIF-2B epsilon and two other eukaryotic translation initiation factors, eIF-4 gamma and eIF-5, and may likewise be involved in the interaction of eIF-2B, eIF-4 gamma and eIF-5 with eIF-2 [11]. Therefore, this eIF5C domain in Ha-eIF5C implies that Ha-eIF5C might also function as a novel translation initiation factor.

Leucine zipper motifs are protein-protein dimerization motifs consisting of heptad repeats of leucine residues that form a coiled-coil structure [12]. These motifs have been well described in the context of transcription factors such as c-Fos and c-Jun, where they mediate homo- and hetero-dimerization critical for the DNA binding properties of these transcription factors [13]. Proteins containing leucine zipper motif have been reported to be related with larval growth, molting and metamorphosis in *D. melanogaster *[14,8]. Our work provides evidence that the expression of Ha-eIF5C, which contains a leucine zipper motif, is upregulated during metamorphosis. We speculate that through its leucine zipper domain, Ha-eIF5C may be involved in transcriptional regulation during insect development.

The expression profile of Ha-eIF5C was correlated with the metamorphic process of *H. armigera*. In our study, *Ha-eIF5C *was upregulated during metamorphosis in the head-thorax, integument, midgut and fat body. Thummel [15] reported that apoptotic and autophagic programmed cell death pathways are involved in tissue histolysis and remodeling during metamorphosis. Gorski *et al*. [16] confirmed that programmed cell death in the salivary glands of *D. melanogaster *requires active protein synthesis, even though cell death is a degradative cellular process. Gorski *et al*. [16] also found significant upregulation of several translation-initiation factors.

Molting and metamorphosis of larvae are very important physiological behaviors in insects, and are governed by two hormones, namely, 20E and juvenile hormone (JH) [17]. Wang *et al*. [18] showed that *H. armigera *had a similar developmental schedule as compared with *Manduca sexta*. 20E levels increase during the late stages of the final (wandering) instar in *M. sexta *larvae, before pupal ecdysis, and then decrease at the pupal ecdysis [1]. The expression of the *Ha-eIF5C *transcript went with the titer of 20E and was enhanced after being injected with 20E, which suggested that it was regulated by 20E *in vivo*. Moreover, the fact that the expression level of *Ha-eIF5C *in HaEpi cell line decreased after EcR or USP was knocked down demonstrated that *Ha-eIF5C *was upregulated by 20E via EcR or USP transcription factor.

Ha-eIF5C appears localized in both the cytoplasm and the nucleus in the midgut, integument and fat body. However, it was identified as a cytoplasmic protein in *D. melanogaster *[10]. In light of its function in the initiation phase of protein synthesis, eIFs were often targeted in the cytoplasm. However, some eIFs such as eIF4E are distributed in the cytoplasm and the nucleus. In the cytoplasm, eIF4E acts in the rate-limiting step of translation initiation. In the nucleus, eIF4E facilitates the nuclear export of a subset of mRNAs. Both of these functions contribute to eIF4E's ability to oncogenically transform cells [19]. Neither eIF5C nor eIF4E contain classical nuclear localization signals (NLSs) predicted by the bioinformatics method . They might act in consonance with some assistant factors that are imported into the nucleus. Dostie *et al*. [20] demonstrated that eIF4E-Transporter (4E-T) is a nucleocytoplasmic shuttling protein that contains an eIF4E-binding site, one bipartite NLS and two leucine-rich nuclear export signals, which mediate the nuclear import of eIF4E via the importin αβ pathway by a piggy-back mechanism.

## Conclusion

Ha-eIF5C possibly functions as a novel translation initiation factor in protein synthesis just like eIF5C of *D. melanogaster*. However, it was interesting to find that Ha-eIF5C was upregulated during metamorphosis. Likewise, it was equally interesting to discover that the expression of *Ha-eIF5C *transcript was enhanced by 20E through EcR and USP. Thus, we hypothesize that Ha-eIF5C possibly functions as a regulator of cotton bollworm development, in addition to its role as a translation initiation factor.

## Methods

### Insects

The larvae of the cotton bollworm were maintained in this laboratory with an artificial diet described by Zhao *et al. *at 28°C under a light:dark ratio of 14:10 h [21]. Moths were fed with 2% sugar water.

### Molecular cloning of Ha-eIF5C gene

A fragment of *Ha-eIF5C *was obtained by suppression subtractive hybridization (SSH) using the metamorphically committed larvae (6th-72, 96 and 120 h) as the tester and the feeding 5th instar larvae (5th-24 h) as the driver [4]. The full-length cDNA was cloned using the cDNA library of *H. armigera *as a template. The 3' end of the gene was amplified using a gene-specific forward primer, eIF5CF (5'-aactccagcaagggcaagatg-3') and a T7 primer (5'-taatacgactcactataggg-3'). Similarly, the 5' end of the cDNA was amplified by a T3 primer (5'-aattaaccctcactaaaggg-3') and a reverse gene-specific primer eIF5CR (5'-tcttcttcggcgctctgtagc-3').

### Sequence analysis

Similarity analysis was performed by BLASTX . Gene translation and prediction of the deduced protein were performed by ExPASy Proteomics Server , including compute pI/Mw, TMpred, NetPhos, NetNGlyc and NetOGlyc. Signal sequence and motif prediction utilized SMART . Alignments were performed with ClustalW  and GENDOC computer programs .

### Recombinant expression and purification

A pair of primers (eIF5CexpF: tactca**ggatcc**atgagtcagaaggtagaaaaac; eIF5CexpR: tactca**gtcgac**ctaatcttcttcttcgccactc) were designed to amplify the sequence coding for Ha-eIF5C protein (bold indicates *Bam*H I and *Sal *I sites, respectively). The DNA fragment was cut with *Bam*H I and *Sal *I, ligated into expression vector pGEX-4T-1 and transformed into competent *Escherichia coli *BL21 host cells. The recombinant expression of Ha-eIF5C was induced by 0.1 mM Isopropyl β-D-1-Thiogalactopyranoside (IPTG). Thereafter, the cells were centrifuged (6000 g, 10 min), resuspended with Phosphate-Buffered Saline (PBS, 140 mM NaCl, 2.7 mM KCl, 10 mM Na_2_HPO_4 _and 1.8 mM KH_2_PO_4_, pH 7.4) containing 0.1% Triton X-100 and sonicated. The recombinant GST-eIF5C was expressed in supernatant and purified by Glutathione Sepharose 4B.

### Antiserum preparation

Rabbit polyclonal antiserum against Ha-eIF5C was prepared using recombinant protein purified from *E. coli *by SDS-PAGE. About 200 μg protein was diluted with saline and mixed with the same volume of complete Freund's adjuvant. It was then injected hypodermically into the back of the rabbit. After three weeks, the emulsified mixture of 200 μg purified recombinant protein and incomplete Freund's adjuvant was then subcutaneously injected into the rabbit. Two weeks later, the rabbit was given booster injections of 500 μg antigen without adjuvant and the antiserum samples were collected. The specificity of the antiserum was examined by immunoblotting and the antiserum was used in all the immunoassay experiments.

### Immunoblotting

We followed previously reported procedures [22]. Protein extracts (100 μg) of the *H. armigera *tissues were separated using 12.5% SDS-PAGE and transferred onto a nitrocellulose membrane. Antiserum against Ha-eIF5C was diluted 1:100 in 2% non-fat milk in Tris-buffered saline (TBS, 10 mM Tris-HCl, pH 7.5 and 150 mM NaCl) and the second antibody of Horseradish Peroxidase (HRP) conjugated to goat anti-rabbit IgG was diluted 1:10,000 in the same blocking buffer (2% non-fat milk in TBS).

### Quantitative real-time PCR analysis

Total RNA was isolated from the head-thorax, integument, midgut, fat body and haemocytes at different developmental stages using Unizol reagent according to the manufacturer' s protocol (Biostar, Shanghai, China). A total of 5 μg RNA was used to reverse transcribe the first strand cDNA (First Strand cDNA Synthesis Kit, MBI Fermentas, St. Leon-Rot, Germany). It was subsequently used as a template in the PCR reactions.

SYBR green-based quantitative real-time PCR (Q-PCR) analysis was performed using PTC-200 DNA Engine thermal cycler (MJ Research) and chromo4 four-color real-time detector (Bio-Rad, America). The following primers were used to amplify a specific fragment of 102 bp: eIF5CF1 (5'-tatggcaatgtgtgatgtcccgtg-3'); eIF5CR1 (5'-cagccaacagcggcgtgtaatg-3'). A 150 bp fragment of β-actin was also amplified as control, with the primers actinF (5'-cctggtattgctgaccgtatgc-3') and actinR (5'-ctgttggaaggtggagagggaa-3'). Amplification conditions were 95°C, 2 min; 40 cycles (95°C, 15 s; 62°C, 50 s; incubated at 72°C for 2 s; plate read; incubated at 82°C for 2 s; plate read); melting curve from 60°C to 95°C, read every 0.5°C, hold 1 s. The data provided from real-time PCR instrumentation were then prepared for input into Microsoft Excel and analyzed using the 2^-ΔCT ^method [23].

### Hormonal regulation of Ha-eIF5C

The 6th instar 0 h larvae (6th-0 h) were injected with steroid 20E (500 ng/larva). 20E was first dissolved to 10 mg/ml in dimethyl sulphoxide (DMSO) and then diluted into 0.1 mg/ml with PBS when injecting worms. Untreated controls were only injected by equivalent amounts of carrier. Total RNA of the midgut was extracted from the injected worms at different developmental periods. A comparison of the differences between the control and the challenged was done by RT-PCR with gene specific primers: eIF5CF1 (5'-tatggcaatgtgtgatgtcccgtg-3'); eIF5CR (5'-tcttcttcggcgctctgtagc-3'). The following procedure was employed: one cycle (94°C, 2 min); 26 cycles (94°C, 30 s; 53°C, 45 s; 72°C, 45 s), followed by a last cycle (72°C, 10 min). The β-actin gene was used for normalization. Each experiment was repeated three times independently. Ratios of Ha-eIF5C to β-actin were calculated with Quantity One (Bio-Rad, Hercules, CA, USA).

### RNAi

The primers of EcRRNAiF1 (5'-gcgtaatacgactcactataggcgctggtataacaacggagga-3') and EcRRNAiR1 (5'-gcgtaatacgactcactataggagctggagacaactcctcacg-3'), EcRRNAiF2 (5'-cgctggtataacaacggagga-3') and EcRRNAiR2 (5'-agctggagacaactcctcacg-3'), USPRNAiF1 (5'-gcgtaatacgactcactataggcgaaccatcccctaagtggttc-3') and USPRNAiR1 (5'-gcgtaatacgactcactataggccttgatgagcaggatctggtc-3'); USPRNAiF2 (5'-cgaaccatcccctaagtggttc-3') and USPRNAiR2 (5'-ccttgatgagcaggatctggtc-3'); GFPRNAiF1 (5'-gcgtaatacgactcactataggtggtcccaattctcgtggaac-3') and GFPRNAiR1 (5'-gcgtaatacgactcactataggcttgaagttgaccttgatgcc-3'); GFPRNAiF2 (5'-tggtcccaattctcgtggaac-3') and GFPRNAiR2 (5'-cttgaagttgaccttgatgcc-3') were used for PCR to amplify the gene fragments. PCR products were purified using a PCR purification kit. dsRNA was synthesized using the MEGAscript™ RNAi kit (Ambion Inc, Ausdin, USA). The procedures of culturing HaEpi cell line and RNAi were performed according to Shao et al. [24]. The green fluorescence protein (GFP) was used as control.

### Immunohistochemistry

The midguts and integuments adhering with fat bodies were dissected in PBS and fixed for 10 h in 4% paraformaldehyde at 4°C. The tissues were dehydrated with a graded series of ethanol. Protein digestion was performed by incubating with proteinase K (20 μg/ml) for 15 min at 37°C. Sections were blocked in 2% bovine serum albumin (BSA), incubated with a primary antibody against Ha-eIF5C diluted to 1:100, and then with a goat anti-rabbit-ALEXA 488 antibody (Eugene, United States) diluted to 1:1000 in PBS with 2% BSA at room temperature for 30 min. The nuclei were stained with 4'-6-Diamidino-2-phenylindole dihydrochloride (DAPI, 1 μg/mL in water, San Jose, United States) for 10 min. Negative controls were treated in the same manner, but pre-immune rabbit serum was used in place of the antiserum against Ha-eIF5C. Fluorescence was detected with an Olympus BX51 fluorescence microscope.

### Accession numbers

The nucleotide sequence reported in this paper has been submitted to GenBank with accession number [GenBank: EU526835].

## Authors' contributions

DJD performed the study. JXW participated in the design and coordination of the work. XFZ conceived the study and helped to draft the final version of this manuscript. All authors read and approved the final manuscript.

## References

[B1] Riddiford LM, Hiruma K, Zhou X, Nelson CA (2003). Insights into the molecular basis of the hormonal control of molting and metamorphosis from *Manduca sexta *and *Drosophila melanogaster*. Insect Biochem.

[B2] Yin VP, Thummel CS (2005). Mechanisms of steroid-triggered programmed cell death in *Drosophila*. Semin Cell Dev Biol.

[B3] Lee CY, Wendel DP, Reid P, Lam P, Thummel CS, Baehrecke EH (2000). E93 directs steroid-triggered programmed cell death in *Drosophila*. Mol Cell.

[B4] Dong D-J, He H-J, Chai L-Q, Zhao X-F, Wang J-X (2007). Identification of genes differentially expressed during larval molting and metamorphosis of *Helicoverpa armigera*. BMC Dev Biol.

[B5] Rhoads RE (1999). Signal transduction pathways that regulate eukaryotic protein synthesis. J Biol Chem.

[B6] Li J, Li W-X (2006). A novel function of Drosophila eIF4A as a negative regulatior of Dpp/BMP signalling that mediates SMAD degradation. Nature Cell Biology.

[B7] Ji Y, Shah S, Soanes K, Islam MN, Hoxter B, Biffo S, Heslip T, Byers S (2008). Eukaryotic initiation factor 6 selectively regulates Wnt signaling and beta-catenin protein synthesis. Oncogene.

[B8] Hewes RS, Schaefer AM, Taghert PH (2000). The cryptocephal gene (ATF4) encodes multiple basic-leucine zipper proteins controlling molting and metamorphosis in *Drosophila*. Genetics.

[B9] Nishinaka N, Hongo S, Zhou CJ, Shioda S, Takahashi R, Yamauchi Y, Ohashi T, Ohki T, Nakada N, Takeda F, Takeda M (2000). Identification of the novel developmentally regulated gene, Bdm2, which is highly expressed in fetal rat brain. Brain Res Dev Brain Res.

[B10] Wang D-Y, Xu M, Liu J-B, Wan Y-Q, Deng H-T, Dou F, Xie W (2006). *Drosophila *Ecp is a novel ribosome associated protein interacting with dRPL5. Biochim Biophys Acta.

[B11] Koonin EV (1995). Multidomain organization of eukaryotic guanine nucleotide exchange translation initiation factor eIF-2B subunits revealed by analysis of conserved sequence motifs. Protein Sci.

[B12] Landschulz WH, Johnson PF, McKnight SL (1988). The leucine zipper: a hypothetical structure common to a new class of DNA binding proteins. Science.

[B13] Junius FK, O'Donoghue SI, Nilges M, Weiss AS, King GF (1996). High resolution NMR solution structure of the leucine zipper domain of the c-Jun homodimer. J Biol Chem.

[B14] Reddy KL, Rovani MK, Wohlwill A, Katzen A, Storti RV (2006). The Drosophila Par domain protein I gene, Pdp1, is a regulator of larval growth, mitosis and endoreplication. Dev Biol.

[B15] Thummel CS (2001). Steroid-triggered death by autophagy. BioEssays.

[B16] Gorski SM, Chittaranjan S, Pleasance ED, Freeman JD, Anderson CL, Varhol RJ, Coughlin SM, Zuyderduyn SD, Jones SJ, Marra MA (2003). A SAGE approach to discovery of genes involved in autophagic cell death. Curr Biol.

[B17] Hiruma K, Riddiford LM (2001). Regulation of transcriptionfactors MHR4 and bFTZ-F1 by 20-hydroxyecdysone during a larval molt in the tobacco hornworm, *Manduca sexta*. Dev Biol.

[B18] Wang J-L, Jiang X-J, Wang Q, Hou L-J, Xu D-W, Wang J-X, Zhao X-F (2007). membrane protein gene in the midgut of *Helicoverpa armigera *Identification and expression profile of a putative basement. BMC Dev Biol.

[B19] Topisirovic I, Ruiz-Gutierrez M, Borden KL (2004). Phosphorylation of the eukaryotic translation initiation factor eIF4E contributes to its transformation and mRNA transport activities. Cancer Res.

[B20] Dostie J, Ferraiuolo M, Pause A, Adam SA, Sonenberg N (2000). A novel shuttling protein, 4E-T, mediates the nuclear import of the mRNA 5' cap-binding protein, eIF4E. EMBO J.

[B21] Zhao X-F, Wang J-X, Wang Y-C (1998). Purification and characterization of a cysteine proteinase from eggs of cotton boll worm, *Helicoverpa armigera*. Insect Biochem Mol Biol.

[B22] Zhao X-F, An X-M, Wang J-X, Dong D-J, Du X-J, Sueda S, Kondo H (2005). Expression of the *Helicoverpa *cathepsin B-like proteinase during embryonic development. Arch Insect Biochem Physiol.

[B23] Kenneth JL, Thomas DS (2001). Analysis of Relative Gene Expression Data Using Real Time Quantitative PCR and the 2^-ΔΔCT ^Method. METHODS.

[B24] Shao H-L, Zheng W-W, Liu P-C, Wang Q, Wang J-X, Zhao X-F (2008). Establishment of a New Cell Line from Lepidopteran Epidermis and Hormonal Regulation on the Genes. Plos One.

